# Quantification of visceral adipose tissue by computed tomography and magnetic resonance imaging: reproducibility and accuracy

**DOI:** 10.1590/0100-3984.2017.0211

**Published:** 2019

**Authors:** Lorenzo Carlo Pescatori, Edoardo Savarino, Giovanni Mauri, Enzo Silvestri, Maurizio Cariati, Francesco Sardanelli, Luca Maria Sconfienza

**Affiliations:** 1 Università degli Studi di Milano, Milano, Italia.; 2 Università di Padova, Padova, Italia.; 3 Istituto Europeo di Oncologia, Milano, Italia.; 4 Ospedale Evangelico Internazionale, Genova, Italia.; 5 ASST Santi Paolo e Carlo, Presidio San Carlo Borromeo, Milano, Italia.; 6 IRCCS Policlinico San Donato, San Donato Milanese, Milano, Italia.; 7 IRCCS Istituto Ortopedico Galeazzi, Milano, Italia.

**Keywords:** Intra-abdominal fat, Image processing, computer-assisted/methods, Tomography, X-ray computed, Magnetic resonance imaging, Reproducibility of results, Gordura intra-abdominal, Processamento de imagem assistida por computador/métodos, Tomografia computadorizada, Ressonância magnética, Reprodutibilidade dos testes

## Abstract

**Objective:**

To evaluate the feasibility of quantifying visceral adipose tissue (VAT) on computed tomography (CT) and magnetic resonance imaging (MRI) scans, using freeware, as well as calculating intraobserver and interobserver reproducibility.

**Materials and Methods:**

We quantified VAT in patients who underwent abdominal CT and MRI at our institution between 2010 and 2015, with a maximum of three months between the two examinations. A slice acquired at the level of the umbilicus was selected. Segmentation was performed with the region growing algorithm of the freeware employed. Intraobserver and interobserver reproducibility were evaluated, as was the accuracy of MRI in relation to that of CT.

**Results:**

Thirty-one patients (14 males and 17 females; mean age of 57 ± 15 years) underwent CT and MRI (mean interval between the examinations, 28 ± 12 days). The interobserver reproducibility was 82% for CT (bias = 1.52 cm^2^; *p* = 0.488), 86% for T1-weighted MRI (bias = −4.36 cm^2^; *p* = 0.006), and 88% for T2-weighted MRI (bias = −0.52 cm^2^; *p* = 0.735). The intraobserver reproducibility was 90% for CT (bias = 0.14 cm^2^; *p* = 0.912), 92% for T1-weighted MRI (bias = −3,4 cm^2^; *p* = 0.035), and 90% for T2-weighted MRI (bias = −0.30 cm^2^; *p* = 0.887). The reproducibility between T1-weighted MRI and T2-weighted MRI was 87% (bias = −0.11 cm^2^; *p* = 0.957). In comparison with the accuracy of CT, that of T1-weighted and T2-weighted MRI was 89% and 91%, respectively.

**Conclusion:**

The program employed can be used in order to quantify VAT on CT, T1-weighted MRI, and T2-weighted MRI scans. Overall, the accuracy of MRI (in comparison with that of CT) appears to be high, as do intraobserver and interobserver reproducibility. However, the quantification of VAT seems to be less reproducible in T1-weighted sequences.

## Abstract (pt)

**Objetivo:**

Avaliar a viabilidade da quantificação do tecido adiposo visceral (TAV) pela tomografia computadorizada (TC) e ressonância magnética (RM) usando um *software freeware*, e também calcular a reprodutibilidade intraobservador e interobservador.

**Materiais e Métodos:**

Foi quantificado o TAV em pacientes submetidos a TC e RM de abdome em nossa instituição, entre 2010 e 2015, com um intervalo máximo de três meses entre os dois exames. Selecionou-se um corte adquirido ao nível da cicatriz umbilical. A segmentação foi realizada com o algoritmo de crescimento de região do *freeware* utilizado. As reprodutibilidades intraobservador e interobservador foram avaliadas, assim como a acurácia da RM em relação à TC.

**Resultados:**

Trinta e um pacientes (14 homens e 17 mulheres; média de idade: 57 ± 15 anos) realizaram TC e RM (intervalo médio entre os exames: 28 ± 12 dias). A reprodutibilidade interobservador foi 82% para TC (viés = 1,52 cm^2^; *p* = 0,488), 86% para RM ponderada em T1 (viés = −4,36 cm^2^; *p* = 0,006) e 88% para RM ponderada em T2 (viés = −0,52 cm^2^; *p* = 0,735). A reprodutibilidade intraobservador foi 90% para TC (viés = 0,14 cm^2^; *p* = 0,912), 92% para RM ponderada em T1 (viés = −3,4 cm^2^; *p* = 0,035) e 90% para RM ponderada em T2 (viés = −0,30 cm^2^, *p* = 0,887). A reprodutibilidade entre a RM ponderada em T1 e a RM ponderada em T2 foi 87% (viés = −0,11 cm^2^; *p* = 0,957). Em comparação com a TC, a acurácia da RM ponderada em T1 e T2 foi 89% e 91%, respectivamente.

**Conclusão:**

O programa utilizado pode ser usado para quantificar o TAV na TC, na RM ponderada em T1 e na RM ponderada em T2. No geral, a acurácia da RM (em comparação com a TC) parece ser alta, assim como a reprodutibilidade intraobservador e interobservador. No entanto, a quantificação do TAV parece ser menos reprodutível nas sequências ponderadas em T1.

## INTRODUCTION

In the human body, the main function of white adipose tissue is to contribute to energy homeostasis by absorbing and storing lipids, as well as by preventing ectopic lipid deposition. White adipose tissue deposits are found mainly in the subcutaneous compartments of the upper and lower body, as well as in the visceral compartment^(^1^)^. In recent decades, evidence has been mounting that the quantity of visceral adipose tissue (VAT) is linked to a number of metabolic dysfunctions, such as insulin resistance, hyperinsulinemia, dyslipidemia, and hypertension^(^2^)^. In addition, calculating the variation in the quantity of VAT over time can be a useful way of evaluating outcomes in patients who have undergone bariatric surgery^(^3^)^, have dietary restrictions^(^4^)^, participate in weight loss programs, or follow specific physical exercise regimens^(^5^)^.

Various methods have been proposed to calculate the amount of fat tissue *in vivo*. In clinical practice, some anthropometric indices have been proposed for quick, reliable evaluation^(^6^)^, such as waist circumference, hip circumference, waist-to-hip ratio, skinfold thickness, and body mass index (BMI), although none of those are able to differentiate the distribution among the compartments or to distinguish between fat content and muscle mass. Although ultrasound has proven to be an accurate means of evaluating the thickness of subcutaneous fat^(^7^)^, its performance continues to be suboptimal for the quantification of VAT^(^8^)^. To address these issues, some authors have used other tools, such as dual-energy X-ray absorptiometry^(^9^)^ and body impedance analysis^(^10^)^, which provide data on lean and fat tissue. However, quantitative evaluation of body fat distribution is still difficult to perform.

Computed tomography (CT) and magnetic resonance imaging (MRI) have both been used as tools to investigate the distribution of subcutaneous adipose tissue (SCAT) and VAT^(^11^-^13^)^. Although each method shows advantages and disadvantages for that purpose, they both can accurately quantify VAT and SCAT^(^14^)^, thus quantifying total adipose tissue. CT is considered the most well-established imaging method for abdominal fat quantification, because adipose tissue has always the same (low) density. On MRI scans, the signal intensity of fat is high in T1- and T2-weighted sequences, although the numerical value varies depending on several factors^(^15^)^. In the use of CT and MRI, one option is to evaluate the amount of fat contained in a single image (slice) acquired at the level of the umbilicus, which has been reported to correlate well with the total VAT^(^16^)^. The use of that strategy results in considerably less radiation exposure during CT and in a markedly shorter duration of MRI examinations^(^16^)^. However, some limitations of single-slice analysis have also been reported, mainly the fact that VAT can undergo great variations due to bowel movement or variable filling of the intestine^(^17^)^.

Several types of software have been used in the analysis of images obtained from CT and MRI scans. Some such software is developed in-house, and the results are therefore not reproducible, because the software is not publicly available^(^18^)^. Other studies have employed specific plugins that can be used with freeware (e.g., ImageJ; NIH, Bethesda, MD, USA), although such plugins are very difficult to use in clinical practice^(^19^)^. OsiriX (Pixmeo, Geneva, Switzerland) is image processing software, dedicated to medical imaging, that is widely used in many radiological applications. The basic version of OsiriX is available for free online. The region growing algorithm of the software can be used in order to calculate the area of different compartments of the body and has previously been used in abdominal imaging^(^20^)^.

The objective of this study was to test the feasibility of using OsiriX to calculate the amount of VAT in patients who have undergone CT and MRI of the abdomen. We also calculated the intraobserver and interobserver reproducibility.

## MATERIALS AND METHODS

### Study population

This was a retrospective study designed to quantify VAT in patients who underwent abdominal CT and abdominal MRI at our institution between 2010 and 2015. The study was approved by the local institutional review board, and the requirement for written informed consent was waived. The image archive and communication system of our hospital were screened to identify patients who had undergone abdominal CT and abdominal MRI, for any reason, with no more than three months between the two examinations. The exact interval between the CT and MRI examinations was noted. Examinations of the upper abdomen, lower abdomen, or entire abdomen, with or without contrast, were included in the evaluation. For both imaging methods, a slice acquired at the level of the umbilicus was considered. We included CT examinations with a non-contrast acquisition and MRI examinations with at least one non-contrast, non-fat-saturated T1- or T2-weighted sequence.

### Image acquisition: MRI

MRI examinations were performed in one of two 1.5 T MRI scanners (Symphony or Aera; Siemens Healthineers, Erlangen, Germany) equipped with phased-array abdominal coils. Depending on the clinical problem to be investigated, different acquisition sequences were used. However, all of the cases included the acquisition of at least one T1-weighted sequence (breath-hold acquisition; echo time = 4.76 ms; repetition time = 280 ms; number of excitations = 1; matrix, 256 × 256; and slice thickness = 4 mm) or one T2-weighted sequence (breath hold acquisition; echo time = 199 ms; repetition time = 4000 ms; number of excitations = 1; matrix, 256 × 256; and slice thickness = 4 mm). When it was available, we selected the slice acquired at the level of the umbilicus in a non-contrast T1-weighted sequence, a non-contrast T2-weighted sequence, or both.

### Image acquisition: CT

CT examinations were performed in multidetector scanners, either a 16-slice scanner (Somatom Emotion; Siemens Healthineers) or a 64-slice scanner (Somatom Definition; Siemens Healthineers). The technical parameters of CT acquisition were adjusted according to the clinical problem under investigation and patient body size. The slice acquired at the level of the umbilicus was selected in the non-contrast acquisition. The slice thickness was 5 mm.

### Image analysis

For CT and MRI, the images were obtained from the archive at our hospital. Those images were uploaded to a separate workstation on which the OsiriX software was installed.

On CT scans, abdominal fat has a hypodense appearance, whereas it has a high signal intensity on T1- and T2-weighted MRI scans. The individual CT and MRI scans were anonymized and analyzed in random order by two readers, working independently-a radiology resident and a radiologist, both with experience in abdominal imaging (more than two years and more than ten years, respectively). Prior to that analysis, both readers had a training session in which a series of five CT scans and five MRI scans, not included in the study, were evaluated in consensus in order to optimize the segmentation technique.

**Figure 1:**
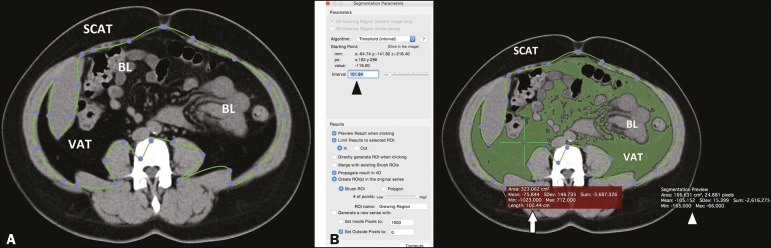
A: Segmentation performed on a CT slice acquired at the level of the umbilicus. The first step was to draw an ROI passing through the abdominal wall, separating the SCAT from the VAT. B: Once the ROI was defined, a point was selected within the VAT area (green cross, white arrow) and an interval of pixels to be taken into account (black arrowhead) was chosen by the reader, in order to include all of the VAT within the ROI in the segmentation process. The software then calculated the segmented area (green area) and assigned it a value (white arrowhead). BL, bowel loop(s).

**Figure 2:**
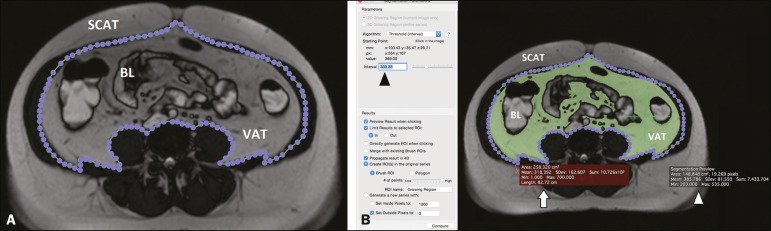
A: Segmentation performed on an MRI slice acquired at the level of the umbilicus. In this case, a true fast imaging T2-weighted sequence was selected. An ROI was drawn to separate the VAT from the SCAT. B: The area within the ROI (white arrow) was calculated by choosing an interval of pixels to be taken into account (black arrowhead). A point within the VAT (green cross) was then selected in order to segment the image. To obtain the VAT area (white arrowhead), the reader has to choose an interval of pixels in order to cover the entire area of adipose tissue surrounding the bowel loops (green area). BL, bowel loop(s).

On each image, a region of interest (ROI) was manually drawn over the abdominal wall to delineate the interface between the abdominal wall and the abdominal fat. No extreme precision is needed in this phase, because the difference in density/intensity between the abdominal wall and the abdominal fat is high on CT and MRI. The region growing (segmentation) algorithm was selected, thus allowing the segmentation ROIs to be drawn with a semi-automated method. The cursor is placed on a portion of the abdominal fat, and the software automatically creates a ROI that includes all pixels with gray levels similar to those selected. The threshold (range of gray levels to be included in the evaluation) can be modified by the operator, who uses a slider to improve the calibration^(^5^,^^21^^)^. At the end of the procedure, the software provides the size of the area included in the region growing algorithm that was considered for statistical analysis. The procedure is depicted in Figures 1 and 2.

### Statistical analysis

The threshold and fat area data are expressed as mean ± standard deviation. For the thresholds, coefficients of variation (CVs) were also calculated.

To evaluate intraobserver reproducibility, the more experienced operator repeated the evaluation, using the same method reported above, after two months. Intraobserver and interobserver reproducibility were assessed with the Bland-Altman method. Values of *p* < 0.05 were considered statistically significant.

In the literature, CT is considered a reliable method to measure VAT. Therefore, CT was used as the reference in our study. The accuracy of MRI was estimated as the inverse consistency error between CT-determined VAT quantity and that measured on T1- and T2-weighted MRI scans.

## RESULTS

During the study period, 4137 patients underwent abdominal CT and 1977 patients underwent abdominal MRI. Among those, there were 31 (14 males and 17 females) who underwent both types of examination. The mean age of the 31 patients was 57 ± 15 years (range, 34-92 years), and the mean interval between the two examinations was 28 ± 12 days. The CT and MRI examinations were performed for a variety of reasons: hepatic lesion (n = 4); pancreatic lesion (n = 5); tumor of the genitourinary tract (n = 7); liver metastases (n = 5); Crohn's disease (n = 5); diverticulitis (n = 1); endometriosis (n = 1); aortic aneurysm (n = 1); gastrointestinal stromal tumor (n = 1); and small bowel lymphoma (n = 1). CT scans were available for all 31 patients; T1-weighted MRI scans were available for 26 patients; T2-weighted MRI scans were available for 23 patients; and T1- and T2-weighted MRI scans were both available for 20 patients.

### Thresholds

As previously described, the threshold (range of gray levels to be included in the evaluation) was adjusted manually. The mean threshold values used for CT scans, T1-weighted MRI scans, and T2-weighted MRI scans in the first evaluation made by the more experienced reader were 145 ± 40 (CV = 27.6%), 475 ± 220 (CV = 46.3%), and 367 ± 159 (CV = 43.3%), respectively.

### Fat area measurement

The mean area of abdominal fat on CT scans, T1-weighted MRI scans, and T2-weighted MRI scans, as calculated by the more experienced reader, was 145 ± 63 cm^2^, 130 ± 71 cm^2^, and 130 ± 68 cm^2^, respectively. The mean area of abdominal fat on CT scans, T1-weighted MRI scans, and T2-weighted MRI scans, as calculated by the less experienced reader, was 143 ± 68 cm^2^, 124 ± 67 cm^2^, and 130 ± 63 cm^2^, respectively.

### Intraobserver and interobserver reproducibility

The intraobserver reproducibility was 90% for CT (bias = 0.14 cm^2^; *p* = 0.912), 92% for T1-weighted MRI (bias = −3.4 cm^2^; *p* = 0.035), and 90% for T2-weighted MRI (bias = −0.30 cm^2^; *p* = 0.887). The interobserver reproducibility was 82% for CT (bias = 1.52 cm^2^; *p* = 0.488), 86% for T1-weighted MRI (bias = −4.36 cm^2^; *p* = 0.006), and 88% for T2-weighted MRI (bias = −0.52 cm^2^; *p* = 0.735). The reproducibility between T1- and T2-weighted MRI was 87% (bias = −0.11 cm^2^; *p* = 0.957).

### Accuracy of MRI

In comparison with that of CT, the accuracy of T1- and T2-weighted MRI was 89% and 92%, respectively.

## DISCUSSION

The main finding of the present study was that OsiriX can be used in order to quantify VAT on CT scans, T1-weighted MRI scans, and T2-weighted MRI scans. Overall, the accuracy of MRI, in comparison with that of CT, was high, as were intraobserver and interobserver reproducibility, although the quantification of VAT seems to be less reproducible on T1-weighted images.

CT and MRI have both been used in order to quantify VAT^(^22^,^^23^^)^, although CT has certainly been used more frequently. Multidetector CT has the advantage of performing quick scans with very high resolution. However, the high dose of ionizing radiation administered to patients is a major limitation of CT. Conversely, MRI has the great advantage of not using ionizing radiation as well as allowing for precise tissue characterization. However, certain contraindications (e.g., non-MRI-compatible implants and claustrophobia), as well as the high cost and long examination times, can limit the use of MRI in clinical practice^(^5^)^. Regarding the evaluation of VAT, one major advantage of CT over MRI is that fat always shows very low attenuation, with little variability among individuals. That allows a relatively narrow threshold to be used when applying a region growing algorithm, as confirmed by our data, given that we found a CV of approximately 25%. That is also why we used CT as the reference to calculate the accuracy of MRI, which was found to be high (approximately 90% for T1- and T2-weighted MRI). One explanation for that finding is that CT and MRI were performed at different time points. Therefore, bowel movement and differences in rectal filling may have affected the quantity of VAT in the selected slice. However, the signal intensity of fat is high on T1- and T2-weighted MRI scans, although that intensity is highly variable, not only among the different types of sequences employed but also among individual patients. That is consistent with our findings, given that the CV exceeded 40% for T1- and T2-weighted MRI scans, which implies that a fully automated system for VAT segmentation and quantification using MRI may be difficult to construct.

Previous studies have quantified VAT on the basis of images of the abdomen as a whole^(^23^)^ or a single slice acquired at the level of the umbilicus^(^17^)^. Although analysis of the entire abdomen certainly has the advantage of greater accuracy, it is extremely time consuming and hardly applicable in clinical practice. Various authors have demonstrated that VAT quantification using a single slice acquired at the level of any one of several anatomic landmarks correlates strongly with total VAT. In a study comparing dual-energy X-ray absorptiometry evaluation of whole-body fat and CT evaluation of SCAT at the level of the interspace of the fourth and fifth lumbar vertebrae (L4-L5), Smith et al.^(^24^)^ found that the two approaches correlated strongly, especially among men. Abate et al.^(^16^)^ found that, although the most reliable single-slice evaluation was achieved with a slice acquired at the L2-L3 level, the addition of a slice acquired at the L1-L2 level and another acquired at the L3-L4 level can increase the predictability from 85% to 90%. Even if the use of three slices is possible, it takes a considerable amount of time to perform the segmentation and the approach should therefore be used only in cases in which greater precision is needed. A slice acquired at the level of the umbilicus has been used because it is commonly included in abdominal examinations performed for any reason. In one study using that approach, Schwenzer et al.^(^17^)^ found that VAT measured at the level of the umbilicus correlated strongly with total VAT, especially among women.

A wide variety of software has been used for VAT quantification, all such software requiring continuous adjustments by the operator. However, most studies on the topic have provided very few technical details, which limits the reproducibility of the results. Addeman et al.^(^15^)^ compared a new automated software known as AdipoQuant with the free software ImageJ, the latter having already been used for this purpose^(^19^)^. The authors found that the two programs provided almost identical VAT values, with excellent agreement. However, the processing time per slice was only 2 s for AdipoQuant, compared with 8 min for ImageJ. OsiriX has previously been used for adipose tissue quantification. In a study involving 62 obese patients, O'Leary et al.^(^25^)^ used OsiriX to quantify VAT, as a means of determining the risk of acute pancreatitis. Those authors found that the quantity of VAT correlated positively with the risk of pancreatitis, although they did not report exactly how segmentation was performed. Kinsella et al.^(^26^)^ also used OsiriX to evaluate changes in fat distribution in patients who underwent renal transplantation, identifying a significant correlation between VAT and BMI, although they also provided no details about the segmentation technique. Lee et al.^(^27^)^ assessed the evaluation of a single CT slice acquired at the level of the umbilicus, as analyzed with the Rapidia software (3DMED, Seoul, Korea), in comparison with the use of bioelectrical impedance analysis, in terms of the quantification of VAT. The authors found that the VAT area was smaller when evaluated by bioelectrical impedance analysis than when evaluated by single-slice CT, with a tendency to increase in parallel with increases in BMI. Yu et al.^(^28^)^ also used Rapidia to evaluate the correlation between VAT and liver fibrosis among patients with nonalcoholic fatty liver disease, finding that the VAT area was significantly larger in the patients with fibrosis than in those without.

In addition to the commercial software and freeware available, in-house systems of VAT quantification have been developed and described by various authors. The main limitations of such studies are that they provide few technical details on the software build and that the software is not publicly available, thus precluding any testing of the reproducibility of the results. Maurovich-Horvat et al.^(^29^)^ performed a semi-automated evaluation of CT-based fat quantification in obese population with in-house software, finding excellent intraobserver and interobserver reproducibility. Yoshizumi et al.^(^30^)^ evaluated VAT in a CT slice acquired at the level of the umbilicus using an in-house algorithm: the attenuation range of CT values for fat tissue was calculated, and a related histogram was constructed, considering the mean attenuation plus or minus two standard deviations. Those authors also found that intraobserver and interobserver reproducibility were high. In our study, intraobserver reproducibility was ≥ 90%, whereas interobserver reproducibility was lower, although still relatively high (82-88%). We found that statistical significance was achieved only for the T1-weighted MRI scans. This somewhat unexpected finding might be explained by the fact that the hyperintense fluid within the bowel-which has a signal intensity similar to fat-could somehow have affected the segmentation.

OsiriX offers several advantages over other software in the evaluation of adipose tissue. First, because it is freeware, there is a greater likelihood that data will be comparable across studies. Second, because it allows rapid data analysis, it can be applied to large populations as well as to several examinations of the same patient in order to analyze changes over time. In addition, we have demonstrated that the VAT segmentation performed with OsiriX has high intraobserver and interobserver reproducibility, which underscores the applicability of this method.

The clinical relevance of the present study mainly resides in the fact that we have shown that it is possible to use MRI as a reliable means of quantifying VAT. The main advantage of that approach is the absence of ionizing radiation, which implies that evaluations can be repeated as needed over time. In addition, MRI is particularly useful in specific cohorts of patients (e.g., those with hepatic lesions, pancreatic lesions, or Crohn's disease). Therefore, concurrent quantification of VAT with no need for a separate examination may represent a further advantage, given that the presence of a high quantity of VAT has been implicated in predisposition to several diseases, as well as in a poor response to several treatments^(^2^,^^3^^)^.

Our study has several limitations. First, it was a prospective evaluation of retrospective data, the CT and MRI examinations having been performed at different time points. Although it seems reasonable to assume that the quantity of VAT would not have changed significantly over a period of three months, such changes could have occurred, which would have affected our evaluation and might explain, at least in part, the differences observed. In addition, we included patients with a wide range of diseases, evaluated with different MRI protocols-some including only the upper abdomen, some including only the lower abdomen, and some including both. However, that limitation is mitigated by the fact that we always evaluated the same slice acquired at the level of the umbilicus, in T1- or T2- weighted sequences, in each patient. Furthermore, the sample size was relatively small. Nevertheless, it was possible to obtain high levels of accuracy and reproducibility.

In conclusion, OsiriX can be used in order to quantify VAT on CT and MRI scans (T1-weighted or T2-weighted). We found that MRI showed high accuracy, in comparison with that of CT, as well as high intraobserver and interobserver reproducibility. However, accuracy, intraobserver reproducibility, and interobserver reproducibility were higher for T2-weighted MRI scans, which might therefore be more suitable for VAT quantification.
